# Supplementary material for: The Systems Biology Research Tool: evolvable open-source software
Source: BMC Syst Biol. 2008 Jun 29;2:55. doi: 10.1186/1752-0509-2-55 (PMC2446383; doi:10.1186/1752-0509-2-55)
Supplement: Additional file 1 — SBRT Archive. An archive of the current version of the Systems Biology Research Tool. [file 1752-0509-2-55-S1.zip › sbrt-1.4.0/doc/developers_guide/api/sbrt/kernel/fba/comp/SlpExCurrComputerV2.html]

SlpExCurrComputerV2


|  |  |  |
| --- | --- | --- |
| **Overview** \| **Package** \| **Class** \| **Use** \| **Tree** \| **Deprecated** \| **Index** \| **Help** | |  |
| **PREV CLASS**   **NEXT CLASS** | **FRAMES**    **NO FRAMES**     **All Classes** |  |
| SUMMARY: NESTED \| FIELD \| CONSTR \| METHOD | DETAIL: FIELD \| CONSTR \| METHOD |  |


---


## sbrt.kernel.fba.comp Class SlpExCurrComputerV2

```
java.lang.Object
  sbrt.kernel.fba.comp.SlpExCurrComputerV2
```

**All Implemented Interfaces:**: ExtremeCurrentComputer

---

``` public class SlpExCurrComputerV2 extends java.lang.Object implements ExtremeCurrentComputer ```

This class is an implementation of an extreme current algorithm. This
algorithm was described in the paper: C. Schilling, D. Letscher and B. O.
Palsson, Theory for systemic definition of metabolic pathways and their use
in intrepreting metabolic function from a pathway-oriented perspective.
*Journal of Theoretical Biology* **203** (2000), pp. 285-301. This
algorithm was intended for computing *extreme pathways*, but it has
been modified here to compute *extreme currents* instead. A brief
mention of extreme currents can be found in the paper cited above.

**Author:**
:   This class was written and documented by Jeremiah Wright while in the
    Wagner lab.

---

**Constructor Summary**

`SlpExCurrComputerV2()`
  
          Constructs a new extreme current computer.


| **Method Summary** | |
| --- | --- |
| `SparseDoubleMatrix<?,java.lang.String>` | `getMatrix(IrrevRxnFluxome fluxome)`             Returns the extreme current matrix for the provided fluxome. |
| `SparseDoubleMatrix<?,java.lang.String>` | `getMatrix(IrrevRxnFluxome fluxome, MutableProgressMonitor pm)`             Returns the extreme current matrix for the provided fluxome. |
| `ProgressMonitor` | `getProgress()`             Returns a progress monitor for this extreme current computer. |
| `int` | `getTotal(IrrevRxnFluxome fluxome)`             Returns the total number of "operations" required to compute the extreme currents of the provided fluxome. |

**Methods inherited from class java.lang.Object**

`clone, equals, finalize, getClass, hashCode, notify, notifyAll, toString, wait, wait, wait`

**Constructor Detail**

### SlpExCurrComputerV2

```
public SlpExCurrComputerV2()
```

:   Constructs a new extreme current computer.


**Method Detail**

### getProgress

```
public ProgressMonitor getProgress()
```

:   Returns a progress monitor for this extreme current computer.

    :   **Specified by:**: `getProgress` in interface `ExtremeCurrentComputer`
    :   **Returns:**: a progress monitor for this extreme current computer.

---


### getTotal

```
public int getTotal(IrrevRxnFluxome fluxome)
```

:   Returns the total number of "operations" required to compute the extreme
    currents of the provided fluxome. The number returned by this method is meant
    for use with progress monitors.

    :   **Specified by:**: `getTotal` in interface `ExtremeCurrentComputer`
    :   **Parameters:**: `fluxome` - the fluxome for which the number of operations will be returned. **Returns:**: the total number of steps required to compute the extreme currents of the provided fluxome.

---


### getMatrix

```
public SparseDoubleMatrix<?,java.lang.String> getMatrix(IrrevRxnFluxome fluxome)
```

:   Returns the extreme current matrix for the provided fluxome.

    :   **Specified by:**: `getMatrix` in interface `ExtremeCurrentComputer`
    :   **Parameters:**: `fluxome` - the fluxome for which all extreme currents will be computed. **Returns:**: the extreme current matrix for the provided fluxome.

---


### getMatrix

```
public SparseDoubleMatrix<?,java.lang.String> getMatrix(IrrevRxnFluxome fluxome,
                                                        MutableProgressMonitor pm)
```

:   Returns the extreme current matrix for the provided fluxome.

    :   **Specified by:**: `getMatrix` in interface `ExtremeCurrentComputer`
    :   **Parameters:**: `fluxome` - the fluxome for which all extreme currents will be computed.: `pm` - the progress monitor to be used during computation of extreme currents. **Returns:**: the extreme current matrix for the provided fluxome. Column variables are reaction names, and each row represents an extreme current.


---


|  |  |  |
| --- | --- | --- |
| **Overview** \| **Package** \| **Class** \| **Use** \| **Tree** \| **Deprecated** \| **Index** \| **Help** | |  |
| **PREV CLASS**   **NEXT CLASS** | **FRAMES**    **NO FRAMES**     **All Classes** |  |
| SUMMARY: NESTED \| FIELD \| CONSTR \| METHOD | DETAIL: FIELD \| CONSTR \| METHOD |  |


---
